# Pediatric Neuromuscular Diseases and Psychosocial Wellbeing: Why We Also Need to Invest in Digital Platforms

**DOI:** 10.3389/ijph.2024.1607460

**Published:** 2024-06-14

**Authors:** Oliver Gruebner, Suzanne Elayan, Martin Sykora, Markus Wolf, Michael von Rhein, Marta Fadda

**Affiliations:** ^1^ Faculty of Health Sciences and Medicine, University of Lucerne, Lucerne, Switzerland; ^2^ Epidemiology, Biostatistics, and Prevention Institute, University of Zurich, Zurich, Switzerland; ^3^ Centre for Information Management, Loughborough University, Loughborough, United Kingdom; ^4^ Department of Psychology, University of Zurich, Zurich, Switzerland; ^5^ Child Development Center, University Children’s Hospital Zurich, University of Zurich, Zurich, Switzerland; ^6^ Institute of Public Health, Università della Svizzera Italiana, Lugano, Switzerland

**Keywords:** accessibility, digital health, Duchenne Muscular Dystrophy, e-health, mental health

## Introduction

Despite the ongoing digital transformation in society, certain population groups remain underserved by these advances. The promise of digital platforms, while groundbreaking in many ways, has fallen short in addressing the unique needs of young individuals and their families diagnosed with neuromuscular disorders (NMDs), including Myasthenia Gravis, Amyotrophic Lateral Sclerosis (ALS), Spinal Muscular Atrophy (SMA), or several types of Muscular Dystrophies [1, 2].

### Support for Patients’ and Family Members’ Mental Health

Duchenne Muscular Dystrophy (DMD), is a hereditary disease, causing progressive loss of muscle strength leading to disability and early death [3]. While medical care is often the focus, “*the psychosocial challenges faced by patients and families remain in the shadows,”* says Dr. med. Hamann, Head of Child, and Adolescent Psychosomatics at Inselspital Bern. The moment of diagnosis represents an emotionally devastating turning point. *“It quickly became clear to us parents that this clinical picture would turn our entire family life upside down”* says Dominik, father of a DMD patient. Parents are confronted with the harsh reality of a significantly reduced life expectancy for their child. Yet, DMD – like most NMDs with childhood onset - is a lifelong journey filled with evolving challenges [4, 5]. Patients’ progressing muscular degeneration leads to increased physical dependence, which places a heavy burden on the mental health of families [6, 7]. Low-barrier access to psychologists who accompany patients and families can reduce the mental health burden. While patient organizations, such as ASRIMM, Duchenne-CH, MGR, or SMG aid in finding psychosocial support and digital repositories, such as Psyfinder are available, access to NMD-related psychological care remains elusive in Switzerland. Therefore, chat bots may become appropriate aids in finding adequate support in the future [8], while we must also look at patient safety [9].

### Sustainment of Daily Functioning

The challenges posed by physical dependence on others can be overwhelming for those affected by NMDs. Depending on the degree of disability, family situation, and personal attitude of patients and families, the burden of care and related stressors can get devastating over time.

In Switzerland, crucial support is offered through Spitex, which provides essential ambulatory care. Families can apply for financial compensation through the IV and many Swiss cantons allow relatives to take on roles as “caregivers” under the Social Security Act, with guidance and support given, e.g., by AsFam. This allows families to be hired as caregivers by their disabled relatives, to receive training, and to care for their loved ones while earning an income. However, tensions may arise if patients seek independence from their parents and therefore may prefer external care. The care-related burden may become overwhelming for the parents as well. “*It quickly became clear to us that we could not care for our 100% dependent sons around the clock all week and needed a break,*” says Sacha, father of two children with DMD. “*Balancing work and family life was difficult at first, as we also have a daughter. We were therefore grateful that our children soon wanted and were able to stay overnight twice a week at a foundation facility with a training and residential home after they started school.*” In the context of physical disabilities, foundations such as MES or Rossfeld offer residential and day care groups and nursing, which may reduce such tensions among family members. Finally, not all cantons offer the same level of support for caregivers, creating disparities in access to resources.

Digital platforms may address these challenges through organizational tools for everyday planning, medication intake management targeted directly at patients, as well as further tools to help mitigate frequent turnover of medical personnel. Apps could provide financial and informational management tools to help caregivers navigate the complex financial landscape associated with NMD care. This empowers caregivers to make informed decisions, reduce financial stress, and ensure that their loved ones receive the care they need without compromising their own financial stability. Apps could also provide educational resources including instructional videos, and expert advice on managing care needs. Finally, apps could help bridging the gap between regions with varying levels of support for caregivers. They could provide information on available services, support networks, and resources specific to each canton as well as highly specific expertise outside one’s local region.

### Social Inclusion

In the context of DMD and other NMDs, the challenges extend to low levels of social integration, including childcare, kindergarten, school, and work environments. Within these domains, there’s a risk that children may find themselves excluded from social activities, particularly when they struggle to keep up with their peers. This lack of alignment can have detrimental effects on their social development, self-esteem, or feelings of exclusion and isolation.

When a special education status ISR is in place, the presence of personal assistance from kindergarten level onwards can be a game-changer in managing daily activities, bridging the gap between educational settings and routine consultations with healthcare providers. Patient organizations also offer much-needed counseling, educational training, and information sessions for schoolchildren to create inclusive settings for teachers and students. Digital peer support platforms can bring families and affected persons together to reduce stigmatization and enhance inclusion right from the early stages of the disease.

### Accessibility and Mobility

The built environment, encompassing public and private buildings, fails to meet the diverse social and healthcare needs of individuals affected by NMDs. Even some healthcare facilities themselves are inaccessible. *“It never ceases to amaze me that during the regular examinations for our sons* […] *an escort must always be present, because in the hospital, for example, there is no equipment whatsoever for the back radiography, such as a hoisting lift, to examine people with muscle disease,”* says Sacha. Yet, the hurdles are not solely structural. Communication barriers come into play as well, with some individuals affected by disabilities facing challenges in verbally expressing their needs due to speech difficulties linked to their condition, fear of stigma, or shame to seek assistance.

In Switzerland, public transport must be fully accessible [10]. However, the reality often does not meet this standard. *“When our sons come home on the weekends, we have to plan it very carefully in advance with the SBB train company.”* continues Sacha, *“Ramps have to be organized to board the train, and an external escort service has to bring them onto the train in Zurich, so that we can then pick them up again at our station.”* This compounds the already intricate web of challenges patients with NMD and their families face.

Opportunities emerge from Smart Cities and Internet of Things (IoT). IoT applications may allow for a more equitable and inclusive urban design and transit options facilitating interaction with the environment *via* sensors and digital devices. Traffic lights may be equipped with an interface or programmed so that they become aware of people with reduced mobility to give them more time to cross the streets, while adequately slowing down motorized individual traffic. Furthermore, elevators can be equipped with sensors so that disabled individuals can use them with their smartphones without having to press buttons. Also, navigation tools should contain options for accessibility routing allowing, e.g., wheelchair users to independently navigate the city or use public transport independently.

## Conclusion

We must collectively acknowledge the untapped potential of digital platforms in supporting patients and families affected by NMDs. To realize this potential, a transdisciplinary and comprehensive approach is needed to encompass the technical aspects alongside the ethical, legal, social, and practical dimensions. The task at hand extends beyond merely bridging the digital divide; it is about ensuring that both digital and physical worlds become genuinely inclusive, reliable, trustworthy, and developed with a steadfast commitment to equity at their core. We believe that such improvements could also serve as an example for very similar challenges regarding children with other disabling neuropediatric disorders and their families.

## Abbreviations

ALS: Amyotrophic Lateral Sclerosis; ASFAM: Assistenz für Familien mit pflegenden Angehörigen, www.asfam.ch; ASRIMM: Association Suisse Romande Intervenant contre les Maladies neuroMusculaires, https://www.asrimm.ch; DMD: Duchenne Muscular Dystrophy; Duchenne-CH: Duchenne Schweiz, https://www.duchenne-schweiz.ch; ISR: Integrierte Sonderschulung in der Verantwortung der Regelschule. For example, ISR regulations of Canton Zurich are available here: https://www.zh.ch/de/bildung/informationen-fuer-schulen/informationen-volksschule/volksschule-schulinfo-besonderer-bildungsbedarf/schulinfo-sonderschulung.html; IV: Invalidenversicherung, https://www.ahv-iv.ch/de/Sozialversicherungen/Invalidenversicherung-IV/Hilflosenentschädigung; NMD: Neuromuscular Disorder; MES: Mathilde Escher Stiftung: https://www.mathilde-escher.ch; MGR: Associazione Malattie Genetiche Rare Svizzera italiana, https://www.malattiegeneticherare.ch; Rossfeld: Stiftung Rossfeld, https://www.rossfeld.ch; SBB: Schweizerische Bundesbahnen, for example, the contact center handicap: https://www.sbb.ch/en/timetable/travel-advice/passengers-with-reduced-mobility/sbb-call-center-handicap.html; SMA: Spinal Muscular Atrophy; SMG: Schweizerische Muskelgesellschaft, https://www.muskelgesellschaft.ch; Spitex: Swiss home care nursing service, Umbrella organization: https://www.spitex.ch; SSPH+: Swiss School of Public Health, https://ssphplus.ch; Psyfinder: https://www.psychologie.ch/psychologensuche.
